# Synthesis and Biological Activity of Manganese (II) Complexes of Phthalic and Isophthalic Acid: X-Ray Crystal Structures of [Mn(ph)(Phen)_2_(H_2_O)]· 4H_2_O, [Mn(Phen)_2_(H_2_O)_2_]_2_(Isoph)_2_(Phen)· 12H_2_O and {[Mn(Isoph)(bipy)]_4_· 2.75biby}_n_(phH_2_ = Phthalic Acid; isoph =      Isophthalic Acid; phen = 1,10-Phenanthroline; bipy = 2,2-Bipyridine)

**DOI:** 10.1155/MBD.2000.275

**Published:** 2000

**Authors:** Michael Devereux, Malachy McCann, Vanessa Leon, Majella Geraghty, Vickie McKee, Jan Wikaira

**Affiliations:** 1 Dublin Institute of Technology Cathal Brugha Street Dublin Ireland; 2 Chemistry Department The National University of Ireland Maynooth Co. Kildare Maynooth Ireland; 3 School of Chemistry The Queen's University Belfast BT9 5AG Ireland; 4 Chemistry Department University of Canterbury Private Bag 4800 Christchurch New Zealand

## Abstract

Manganese(II) acetate reacts with phthalic acid (phH_2_) to give [Mn(ph)]·0.5H_2_O (**1**). Reaction of **1** with 1,10-phenanthroline produces [Mn(ph)(phen)]·2H_2_O (**2**) and [Mn(ph)(phen)_2_(H_2_O)]·4H_2_O (**3**). Reaction of isophthalic acid (isophH_2_) with manganese(II) acetate results in the formation of [Mn(isoph)]·2H_2_O (**4**). The addition of the N,N-donor ligands 1,10-phenanthroline or 2,2'-bipyridine to **4** leads to the formation of [Mn_2_ (isoph)_2_(phen)_3_)]·4H_2_O (**5**), [(Mn(phen)_2_(H_2_O)_2_]_2_(isoph)_2_(phen)·12H_2_O (**6**) and {[Mn(isoph)(bipy)]_4_·2.75 biby}_n_ (**7**), respectively. Molecular structures of **3**, **6** and **7** were determined crystallographically. In **3** the phthalate ligand is bound to the manganese via just one of its carboxylate groups in a monodentate mode with the remaining coordination sites filled by four phenanthroline nitrogen and one water oxygen atoms. In **6** the isophthalates are uncoordinated with the octahedral manganese center ligated by two phenanthrolines and two waters. In **7** the Isophthalate ligands act as bridges resulting in a polymeric structure. One of the carboxylate groups is chelating a single manganese with the other binding two metal centres in a bridging bidentate mode. The phthalate and isophthalate complexes, the metal free ligands and a number of simple manganes salts were each tested for their ability, to inhibit the growth of *Candida albicans*. Only the “metal free” 1,10-phenanthroline and its manganese complexes were found to be active.


### SYNTHESIS AND BIOLOGICAL ACTIVITY OF MANGANESE (II) COMPLEXES OF PHTHALIC AND ISOPHTHALIC ACID: X-RAY CRYSTAL STRUCTURES OF $[Mn(ph)(Phen)_2(H_2O)]\cdot4H_2O$, $[Mn(Phen)_2(H_2O)_2]_2(Isoph)_2(Phen)\cdot12H_2O$ AND $\{[Mn(Isoph)(bipy)]_4\cdot2.75bipy\}_n$ ($phH_2$ = PHTHALIC ACID; isoph = ISOPHTHALIC ACID; phen = 1,10-PHENANTHROLINE; bipy = 2,2-BIPYRIDINE)

Michael Devereux*[1], Malachy McCann[2], Vanessa Leon[1], Majella Geraghty[2], Vickie McKee[3] and Jan Wikaira[4]

[1] Dublin Institute of Technology, Cathal Brugha Street, Dublin, Ireland
[2] Chemistry Department, The National University of Ireland Maynooth, Maynooth, Co. Kildare, Ireland
[3] School of Chemistry, The Queen's University, Belfast BT9 5AG, N. Ireland.
[4] Chemistry Department, University of Canterbury, Private Bag 4800, Christchurch, New Zealand.

**Abstract**
Manganese(II) acetate reacts with phthalic acid ($phH_2$) to give $[Mn(ph)]\cdot0.5H_2O$ (**1**). Reaction of **1** with 1,10-phenanthroline produces $[Mn(ph)(phen)]\cdot2H_2O$ (**2**) and $[Mn(ph)(phen)_2(H_2O)]\cdot4H_2O$ (**3**). Reaction of isophthalic acid ($isophH_2$) with manganese(II) acetate results in the formation of $[Mn(isoph)]\cdot2H_2O$ (**4**). The addition of the N,N-donor ligands 1,10-phenanthroline or 2,2'-bipyridine to **4** leads to the formation of $[Mn_2(isoph)_2(phen)_3)]\cdot4H_2O$ (**5**), $[(Mn(phen)_2(H_2O)_2]_2(isoph)_2(phen)\cdot12H_2O$ (**6**) and $\{[Mn(isoph)(bipy)]_4\cdot2.75bipy\}_n$ (**7**), respectively. Molecular structures of **3**, **6** and **7** were determined crystallographically. In **3** the phthalate ligand is bound to the manganese via just one of its carboxylate groups in a monodentate mode with the remaining coordination sites filled by four phenanthroline nitrogen and one water oxygen atoms. In **6** the isophthalates are uncoordinated with the octahedral manganese center ligated by two phenanthrolines and two waters. In **7** the Isophthalate ligands act as bridges resulting in a polymeric structure. One of the carboxylate groups is chelating a single manganese with the other binding two metal centres in a bridging bidentate mode. The phthalate and isophthalate complexes, the metal free ligands and a number of simple manganes salts were each tested for their ability to inhibit the growth of *Candida albicans*. Only the "metal free" 1,10-phenanthroline and its manganese complexes were found to be active.

*Keywords*: crystal structures; phthalic acid; isophthalic acid; 1,10-phenanthroline; bipyridine; metal complexes; *Candida albicans*.

### Introduction
The yeast *Candida albicans* is a commensal of the human body and is considered to be an important fungal pathogen. Oppurtunistic infection can lead to the development of vaginal candidosis (thrush), a condition from which over 75% of women suffer at some stage in their lifetime. Systemic candidosis is often fatal in immunocompromised patients.[1-4] The state-of-the-art drugs currently used to treat *Candida* infections are often ineffective because of problems with resistence or toxicity and subsequently the search for novel anti-fungal agents has gathered momentum.[5] A number of publications has appeared in the literature highlighting the fungicidal activity of novel transition metal complexes.[6] Recently, we have shown that a range of carboxylate complexes icorporating Copper, Manganese and Cobalt metal centres inhibit the growth of *Candida albicans* .[7-11] In the present paper we report the synthesis and structural characterisation of three new manganese(II) complexes derived from either phthalic or isophthalic acid. In addition the *in-vitro* anti-*Candida* activities of the compounds are discussed.

### Materials and Methods
Chemicals were purchased from commercial sources and used without further purification. Infrared spectra were recorded in the region 4000-400 $cm^{-1}$ on a Nicolet-400 Impact spectrometer. Magnetic susceptibility measurements were made using a Johnson Matthey Magnetic Susceptibility balance. $[HgCo(SCN)_4]$ was used as a reference. Conductivity readings were obtained using a Ciba Corning model check-mate 90 conductivity meter. Satisfactory microanalytical data for the complexes were reported by the Microanalytical Laboratory, University College Cork, Ireland.

**Table 1**: Summary of crystal data, data collection, structure solution and refinement details for complexes **3**, **5** and **7**.

| | **Complex (3)** | **Complex (6)** | **Complex (7)** |
|---|---|---|---|
| Complex | $[Mn(ph)(phen)_2(H_2O)]\cdot 4H_2O$ | $[Mn(phen)_2(H_2O)_2]_2(isoph)_2(phen)\cdot 12H_2O$ | $\{[Mn(isoph)(bipy)]_4\cdot 2.75bipy\}_n$ |
| Formula | $C_{32}H_{30}MnN_4O_9$ | $C_{76}H_{80}Mn_2N_{10}O_{24}$ | $C_{99.50}H_{69.90}Mn_4N_{13.50}O_{16}$ |
| Description | yellow triangular prism | pale yellow block | pale yellow plate |
| Crystal size (mm) | 0.625 × 0.35 × 0.20 | 0.46 × 0.22 × 0.12 | 0.37 × 0.33 × 0.02 |
| Temperature (K) | 153(2) | 163(2) | 158(2) |
| *a* (Å) | 11.509(3) | 16.356(2) | 13.419(5) |
| *b* (Å) | 11.990(2) | 20.573(3) | 22.246(8) |
| *c* (Å) | 13.157(2) | 23.304(3) | 30.217(10) |
| $a,\beta,\gamma$ (°) | 69.096(15),70.957(13), 65.074(18) | 90, 106.895(4), 90 | 90, 96.647(6), 90 |
| $U$ (Å$^{-3}$) | 1504.7(5) | 7503(2) | 8960(5) |
| F(000) | 694 | 3392 | 3956 |
| Crystal system | Triclinic | Monoclinic | Monoclinic |
| Space group | P-1 | $P2_1/c$ | P2(1)/n |
| *Z* | 2 | 4 | 4 |
| Density calc. (Mg/m$^3$) | 1.478 | 1.441 | 1.431 |
| $\mu$ (mm$^{-1}$) | 0.502 | 0.423 | 0.627 |
| $T_{max}$, $T_{min}$ | 0.8848, 0.8299 | 1.000, 0.874 | 0.697, 1.000 |
| θ range (°) | 2.20 to 25.00° | 2.37 to 26.41 | 1.97 to 24.00° |
| Index ranges | -12≤h≤0<br>-13≤k≤12<br>-15≤l≤14 | -17≤h≤20<br>-24≤k≤25<br>-27≤l≤11 | -15≤h≤14<br>-25≤k≤20<br>-34≤l≤15 |
| Reflections collected | 5455 | 32135 | 45429 |
| Independent reflections | 5173 | 14452 | 14060 |
| $R_{int}$ | 0.0211 | 0.0427 | 0.0765 |
| Data/restraints/ parameters | 5173 / 0 / 415 | 14452 / 0 / 1009 | 14060 / 142/ 1201 |
| GooF on $F^2$ | 1.062 | 0.854 | 0.679 |
| R1, wR2 [$I > 2\sigma(I)$] | 0.0444, 0.0973 | 0.0429, 0.0915 | 0.0491, 0.1188 |
| R1, wR2 [all data] | 0.0684, 0.1068 | 0.1025, 0.1005 | 0.1663, 0.1369 |
| Deposition No.s | CCDC 151284 | CCDC 151285 | CCDC 151286 |

The crystal data for complexes **3**, **6** and **7** are summarised in Table 1. Data sets for $[Mn(ph)(phen)_2(H_2O)]\cdot 4H_2O$ (**3**) were collected on a Siemens P4 diffractometer whereas those for $[(Mn(phen)_2(H_2O)_2]_2(isoph)_2(phen)\cdot 12H_2O$ (**6**) and $\{[Mn(isoph)(bipy)]_4\cdot 2.75bipy\}_n$ (**7**) were collected using a Siemens SMART CCD diffractometer. All the data for the structures were corrected for Lorentz, polarization and absorption effects. All three structures were solved by Direct methods and refined by full matrix least-squares on $F^2$ using all of the data. All the non-hydrogen atoms were refined with anisotropic atomic displacement parameters and hydrogen atoms bonded to carbon atoms were inserted at calculated positions. For **3** and **6** Hydrogen atoms bonded to oxygen were located from difference Fourier maps and not further refined. In **6** one of the solvate water molecules was disordered and refined with partial occupancy of two sites (O16W 25%, O16' 75%), the hydrogen atoms associated with this molecule were not inserted. As described below, the structure of **7** consists of a polymeric chain with the uncoordinated bipy groups occupying voids in the lattice. The structure can be solved and the polymeric chain refined in a smaller cell ($U$ = 4480 Å$^{-3}$, space group C2/c, $R_{int}$ = 0.042) but we could not satisfactorily model the lattice bipy using this cell. In the larger, $P2_1/n$ cell, uncoordinated bipy molecules were located at three independent sites, two of these were refined without difficulty but some constraints were applied to the third which was refined with 75% occupancy using regular hexagonal geometry for the two six-membered rings. The two nitrogen atoms were not clearly distinguished in the third bipy molecule and all the atoms were refined as carbon. It seems probable that the lattice bipy molecules render the structure formally incommensurate.

All the non-hydrogen atoms were refined with anisotropic atomic displacement, hydrogen atoms were inserted at calculated positions with their isotropic displacement parameters riding on $U_{ij}$ of their carrier atoms (except for those on the partial occupancy bipy molecule, which were not included).

All programs used in the structure solution and refinement are contained in the SHELXL97 package.[12]

**[Mn(ph)]·0.5H$_2$O (1)**: To a solution of phH$_2$ (1.25 g, 7.52 mmol) in ethanol (100cm$^3$) was added manganese (II) acetate (2.00 g, 8.25 mmol), and the mixture was refluxed for 2h. The product formed as a colourless solid. The reaction mixture was allowed to cool to room temperature and the product was filtered off. The compound was washed with two portions of ethanol and then dried in air. Yield: 1.73 g (92.12%). Complex **1** was soluble in dist. H$_2$O, partially soluble in warm ether and insoluble in EtOH, MeOH, acetone and chloroform. Found: C, 42.40;H, 1.92. Calc: C, 42.13;H, 2.21%. IR: 3408.96, 3073.13, 1555.22, 1488.06, 1420.22, 1360.45, 1259.70, 1145.52, 1091.79, 1051.49, 950.75, 856.32, 829.85, 807.45, 735.06, 694.75, 648.51, 594.78 cm$^{-1}$. $\mu_{eff}$ = 5.91 B.M. $\Lambda_M$ (H$_2$O) = 116.21 Scm$^2$mol$^{-1}$.

**[Mn(ph)(phen)]·2H$_2$O (2) and [Mn(ph)(phen)$_2$(H$_2$O)]·4H$_2$O (3)**: Complex **1** (0.5 g, 2.19 mmol) and 1,10-phenanthroline (0.81 g, 4.08 mmol) were refluxed in ethanol (50 cm $^3$) for 2h. Upon cooling to room temperature the resulting suspension was filtered off to give a yellow powder and a yellow solution. The yellow product (complex **2**) was filtered off and then air-dried. Yield: 0.38 g, (40.18%). Complex **2** was partially soluble in warm dist. H$_2$O, EtOH, MeOH, acetone, ether and chloroform. Found: C, 55.5;H, 3.52;N, 6.48. Calc: C, 55.18;H, 3.70;N, 6.43%. IR: 3408.96, 3073.13, 1548.51, 1514.93, 1407.46, 1145.52, 1111.94, 1085.07, 856.72, 769.40, 729.10, 695.52, 648.51 cm$^{-1}$. $\mu_{eff}$ = 5.94 B.M. $\Lambda_M$ ( H$_2$O) = 56.50 Scm$^2$mol$^{-1}$.

The yellow filtrate was set aside to evaporate slowly. Upon standing at room temperature for a week complex **3** deposited as yellow crystals. The crystals were filtered off and air-dried. Yield: 0.38 g, (26.11%). Found: C, 59.81; H, 4.04; N, 8.57. Calc: C, 57.40; H, 4.52; N, 8.37%. IR: 3415.24, 3045.79, 1608.30, 1574.71, 1507.54, 1420.22, 1379.91, 1218.70, 1144.81, 1097.79, 861.20, 846.73, 761.92, 728.34, 721.75, 636.45 cm$^{-1}$. $\mu_{eff}$= 5.87 B.M. $\Lambda_M$ ( EtOH) = 8.20 Scm$^2$mol$^{-1}$.

**[Mn(isoph)]·2H$_2$O (4)**: To a solution of isophH$_2$ (1.10 g, 6.62 mmol) in ethanol (100 cm$^3$) was added manganese (II) acetate (1.78 g, 7.26 mmol), and the mixture was refluxed for 2h. The product formed as a colourless solid. The reaction mixture was allowed to cool to room temperature and then filtered. The product was washed with two portions of ethanol and dried in air. Yield: 1.64 g (97.12%). Complex **4** was soluble in warm H$_2$O, partially soluble in warm MeOH, and insoluble in EtOH, acetone, ether and chloroform. Found: C, 37.35;H, 2.90. Calc: C, 37.67;H, 3.16%. IR : 3314.48, 2280.60, 1648.60, 1615.67, 1547.84, 1481.34, 1454.48, 1380.60, 1165.67, 1085.07, 1004.48, 944.03, 816.42, 735.82, 695.52, 541.04, 447.01 cm$^{-1}$. $\mu_{eff}$ = 5.74 B.M. $\Lambda_M$ (H$_2$O) = 147.17 Scm$^2$mol$^{-1}$.

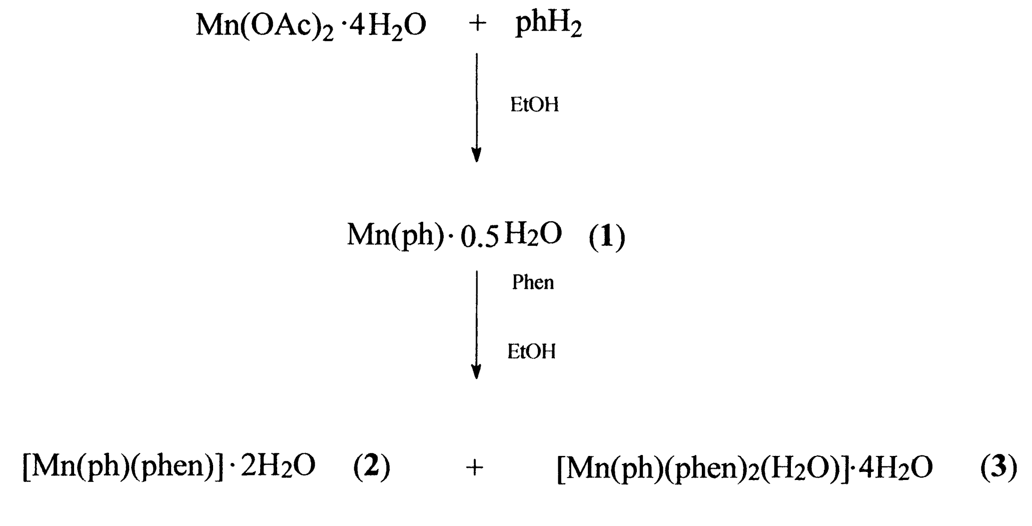

Scheme 1

**[Mn$_2$(isoph)$_2$(phen)$_3$]·4H$_2$O (5) and [Mn(phen)$_2$(H$_2$O)$_2$]$_2$(isoph)$_2$(phen)·12H$_2$O (6)**: Complex **4** (0.5 g, 1.96 mmol) and 1,10-phenanthroline (0.77 g, 3.92 mmol) were stirred for 4h in deionised water (100 cm$^3$). The resulting yellow suspension was filtered yielding a yellow powder and a yellow solution. The powder (Complex **5**) was collected and allowed to dry in air. Yield: 1.02 g (49.54%). Complex **5** was soluble in MeOH and acetone, in warm ether, partially soluble in warm EtOH and chloroform, and insoluble in dist. H$_2$O. Found: C, 58.24;H, 3.14;N, 8.6. Calc: C, 59.43;H, 3.84;N, 8.00%. IR: 3550.00, 3476.12, 3415.24, 3052.51, 1642.54, 1608.30, 1575.37, 1514.26, 1447.76, 1427.61, 1383.63, 1367.161219.40, 1145.52, 1104.50, 1078.36, 856.72, 755.97, 728.34, 641.79 cm$^{-1}$. $\mu_{eff}$ = 5.43 B.M, $\Lambda_M$ (MeOH) = 2.19 Scm$^2$mol$^{-1}$. The yellow filtrate was reduced on a hot plate and subsequently upon standing at room temperature for a week complex **6** deposited as yellow crystals. The crystals were filtered off and air-dried. Yield: 0.12 g, (7.52%). Found: C, 58.16, H, 3.66, N, 9.61. Calc: C, 56.09, H, 4.95, N, 8.61%. IR: 3415.67, 1602.24, 1561.94, 1514.93, 1420.90, 1367.16, 863.43, 843.28, 749.25, 729.10 cm$^{-1}$. $\mu_{eff}$ = 6.16 B.M.

**{[Mn(isoph)(bipy)]$_4$·2.75bipy}$_n$ (7)**: To a solution of **4** (0.51 g, 2.00 mmol) in deionised water (100 cm$^3$) was added 2, 2'-bipyridine (0.92 g, 5.89 mmol) and the mixture was stirred for 4h. The resulting green solution gave yellow crystals (complex 7) after standing at room temperature for one month. The crystals were filtered off, washed with two small portions of water and then allowed to dry in air. Yield: 0.41 g (29.51%). Complex 7 was partially soluble in warm ether and insoluble in dist, $H_2O$, EtOH, MeOH, acetone and chloroform. Found: C, 61.46, H, 3.64, N, 9.73. Calc: C, 61.92, H, 3.63, N, 9.79%. IR: 3402.24, 3059.70, 1662.69, 1602.24, 1561.94, 1475.70, 1441.04, 1432.67, 1380.60, 1320.15, 1273.13, 1246.27, 1158.96, 1098.51, 1058.21, 1017.91, 917.16, 776.12, 755.97, 722.39 $cm^{-1}$. $\mu_{eff}$ = 5.81 B.M. $\Lambda_M$ (Ether) = 0 $Scm^2mol^{-1}$.

**Biological Studies**

*Candida albicans* isolate was obtained commercially from Oxid Culti-loops (ATCC 10231) .The isolate was stored on Sabouraud dextrose agar (SDA) plates at 4 °C.

Suspensions of the test complexes were prepared by grinding them to a fine powder (0.02 g) followed by addition to sterile distilled water (100 $cm^3$). This process yielded stock suspensions of 200 $\mu g\ cm^{-3}$. These suspensions were furthered diluted to 100 $\mu g\ cm^{-3}$.

*Susceptibility testing Method:* RPMI-1640 broth medium (Sigma R 7755) was used for the anti-*Candida* susceptibility testing. The medium (1 $dm^3$) was supplemented with L-glutamine (0.3 g) and morpholinepropanesulfonic acid (MOPS) (34.6 g) and was then adjusted to pH 7.0 using sterile NaOH (0.2 M). The broth microdilution reference method was used NCCLS publication M27-P.[13] Prior to testing, yeast cells were grown on Sabouraud dextrose agar (SDA) at 37 °C for 24 h. Cell suspensions were prepared in sterile phosphate buffered saline (5 $cm^3$) to a density of 1 McFarland standard. A 1:100 dilution of these cell suspensions were made in RPMI-1640 medium so that the cell concentration of the final inoculum was 3.5 × $10^4$ – 5.0 × $10^5$ cells $cm^{-3}$. The prepared cells suspension (900 μl) was dispensed into sterile test tubes and to this was added the test complex solutions (100μl) to yield working solutions of the test complexes of concentration 10 $\mu g\ cm^{-3}$. The test tubes were then incubated in a shaking water bath for 24 h at 37 ° C with continuous shaking. Each complex was assessed in triplicate and three independent experiments were performed.

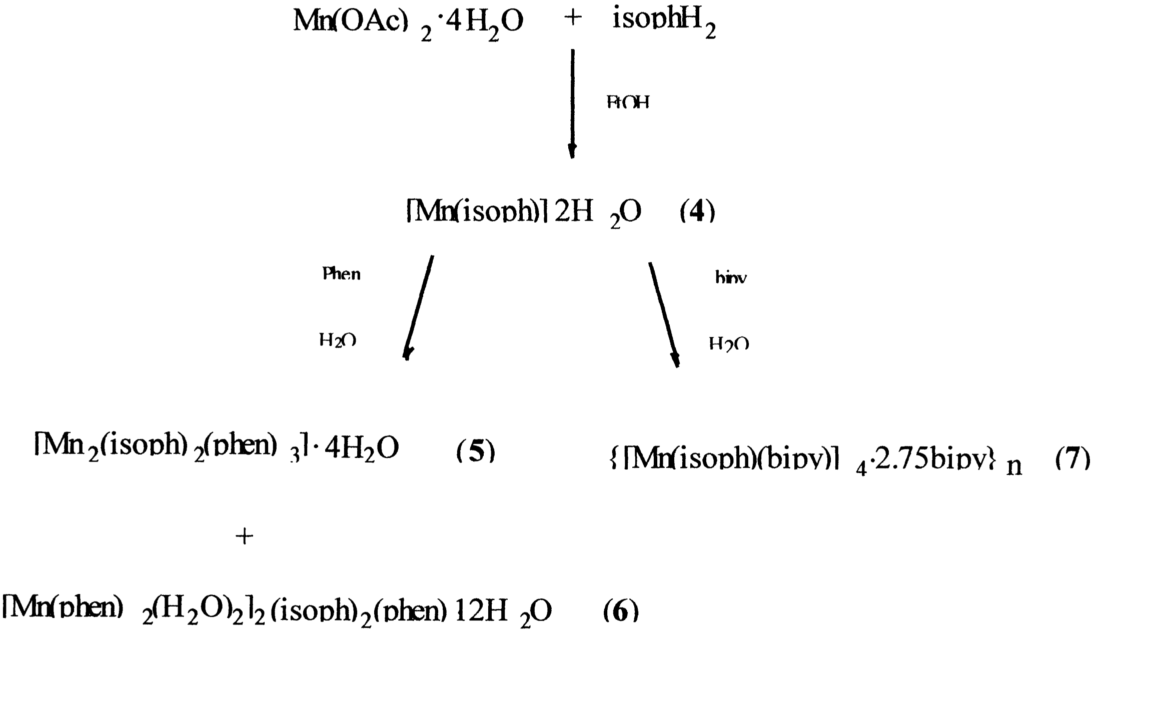

**Scheme 2**

**Results and Discussion**

Reaction pathways to the complexes **1-7** are shown in Scheme 1 and Scheme 2. Manganese(II) acetate reacted smoothly with phthalic acid ($phH_2$) to give [Mn(ph)]·0.5$H_2O$ (**1**) as a colourless powder. Reaction of **1** with 1,10-phenanthroline in ethanol yielded [Mn(ph)(phen)]·2$H_2O$ (**2**) as a yellow precipitate and [Mn(ph)(phen)$_2$($H_2O$)]·4$H_2O$ (**3**) deposited as yellow crystals from the reaction filtrate. Reaction of isophthalic acid ($isophH_2$) with manganese(II) acetate resulted in the formation of [Mn(isoph)]·2$H_2O$ (**4**) as a colourless powder. The addition of the N,N-donor ligands 1,10-phenanthroline or 2,2'-bipyridine to **4** lead to the formation of $[Mn_2(isoph)_2(phen)_3]\cdot 4H_2O$ (**5**), $[(Mn(phen)_2(H_2O)_2]_2(isoph)_2(phen)\cdot 12H_2O$ (**6**) and $\{[Mn(isoph)(bipy)]_4\cdot 2.75bipy\}_n$ (**7**), respectively

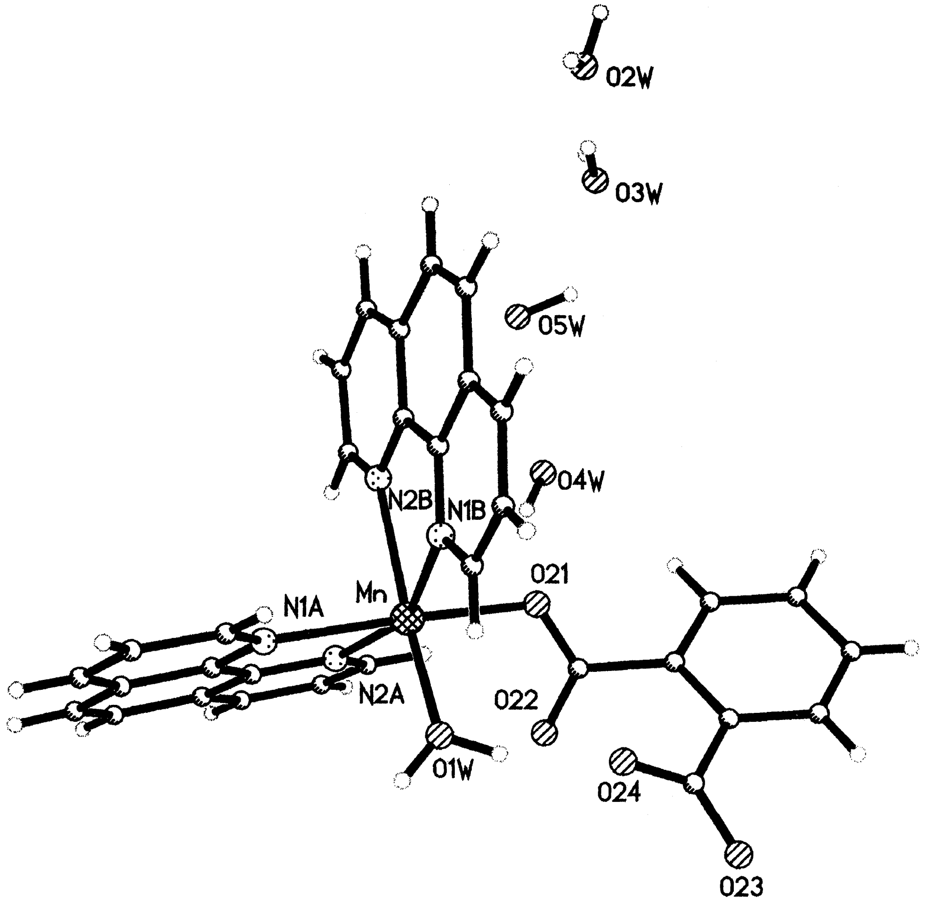

**Figure 1: Structure of $[Mn(ph)(phen)_2(H_2O)]\cdot 4H_2O$ (3)**

The X-ray structure of **3** is shown in Figure 1 and Figure 2 and selected bond lengths and angles for the complex are presented in Table 2. The asymmetric unit consists of $[Mn(ph)(phen)_2(H_2O)]$ (Figure 1). The two phenanthrolines are co-ordinated to the Manganese atom in a bidentate way while the phthalate anion is bound to the metal through only one carboxylate oxygen in a monodentate mode. A water molecule occupies the sixth co-ordination site.

The carboxylate oxygen (O21), the coordinated water oxygen (O1W) and the lattice water oxygens (O2W-O5W) are all involved in Hydrogen bonding (Table 3). The O4W is hydrogen bonded to the co-ordinated carboxylate oxygen (O21) and to another molecule of water (symmetry related). The O5W is hydrogen bonded in the same manner to the upper O3W and to another carboxylate oxygen (symmetry related). The O2W is hydrogen bonded to two different carboxylate groups, and O3W to O2W and the symmetry generated O3W. The co-ordinated water molecule O1W is involved as well in hydrogen bonding to O24 and O23 (symmetry generated). A number of water lattice molecules form columns that run through the middle of the unit cell (Figure 2).

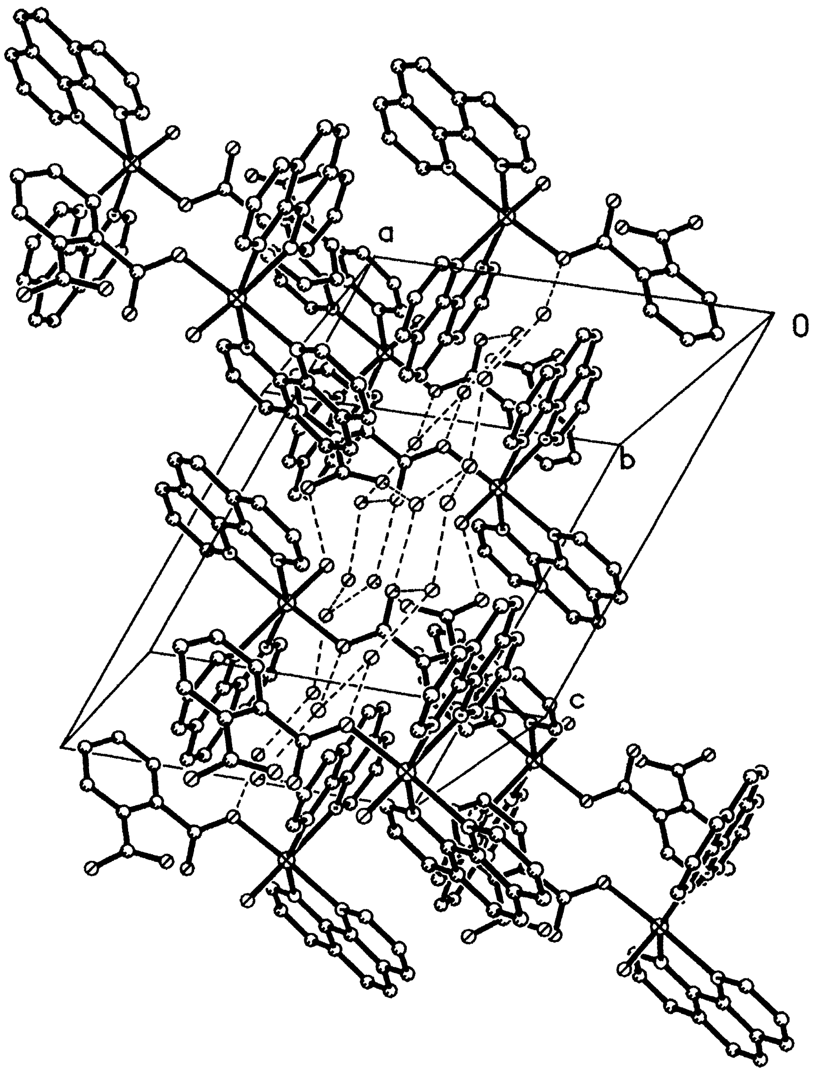

**Figure 2:** The unit cell for $[Mn(ph)(phen)_2(H_2O)]\cdot 4H_2O$ (**3**)

The X-ray structure of **6** is shown in Figure 3 and selected bond lengths and angles for the complex are presented in Table 4. The asymmetric unit consists of two independent $[Mn(phen)_2(H_2O)_2]^{2+}$ cations, two independent isophthalate anions $[isoph]^-$, a non-coordinated phenanthroline molecule and twelve uncoordinated lattice water molecules. (Figure 3)

The two co-ordinated $H_2O$ molecules of each cation are hydrogen bonded (Table 5) to a carboxylate group belonging to one of the isophthalate anions (O1W-O22, O2W-O21, O3W-O31 and O4w-O32).

An important aspect in the crystal is the presence of non-co-ordinate phenanthroline molecule intercalated between two phenanthroline groups on adjacent manganese centres. The non-co-ordinate phenanthroline molecule brings π-π stacking to the crystal, the interplanar distances for the π-π stacking are ca. 3.4Å. The intercalated phenanthroline molecule is also involved in hydrogen bonding (Table 5) to one of the co-ordinated water molecules of each cation (N1E-O3W 2.851, N2E-O2W 2.837Å) (Figure 4). These phenathroline molecules are stacked parallel to the ab plane and perpendicular to the plane containing the phthalate anions (Figure 5).

Table 2. Selected bond lengths [Å] and angles [°] for complex **3**.

| | |
|---|---|
| Mn-O(1W) | 2.097(2) |
| Mn-O(21) | 2.133(2) |
| Mn-N(2A) | 2.265(3) |
| Mn-N(1B) | 2.280(3) |
| Mn-N(1A) | 2.280(3) |
| Mn-N(2B) | 2.311(3) |
| | |
| O(1W)-Mn-O(21) | 92.14(8) |
| O(1W)-Mn-N(2A) | 106.64(9) |
| O(21)-Mn-N(2A) | 103.67(9) |
| O(1W)-Mn-N(1B) | 89.78(9) |
| O(21)-Mn-N(1B) | 90.77(9) |
| N(2A)-Mn-N(1B) | 157.43(9) |
| O(1W)-Mn-N(1A) | 86.96(9) |
| O(21)-Mn-N(1A) | 176.46(9) |
| N(2A)-Mn-N(1A) | 73.34(9) |
| N(1B)-Mn-N(1A) | 92.66(9) |
| | |
| O(1W)-Mn-N(2B) | 162.25(9) |
| O(21)-M .-N(2B) | 90.79(9) |
| N(2A)-Mn-N(2B) | 89.63(9) |
| N(1B)-Mn-N(2B) | 72.68(9) |
| N(1A)-Mn-N(2B) | 91.10(9) |

Table 3. Hydrogen bonds for complex **3** [Å and °].

| D-H...A | d(D-H) | d(H...A) | d(D...A) | <(DHA) |
|---|---|---|---|---|
| O(1W)-H(1WA)...O(24) | 0.93 | 1.85 | 2.760(3) | 164.3 |
| O(2W)-H(2WA)...O(24)#1 | 0.86 | 1.96 | 2.814(3) | 173.9 |
| O(3W)-H(3WA)...O(2W) | 0.98 | 1.77 | 2.730(3) | 166.8 |
| O(2W)-H(2WB)...O(22)#2 | 0.89 | 1.98 | 2.848(3) | 165.0 |
| O(5W)-H(5WB)...O(22)#3 | 0.91 | 1.94 | 2.764(4) | 150.2 |
| O(1W)-H(1WB)...O(23)#4 | 0.91 | 1.72 | 2.625(3) | 173.6 |
| O(4W)-H(4WA)...O(21) | 0.99 | 1.91 | 2.844(3) | 156.3 |
| O(4W)-H(4WB)...O(4W)#3 | 0.77 | 2.09 | 2.783(6) | 149.4 |
| O(3W)-H(3WB)...O(3W)#5 | 0.85 | 1.94 | 2.795(6) | 174.6 |
| O(5W)-H(5WA)...O(3W) | 0.94 | 2.03 | 2.683(4) | 125.4 |

Symmetry transformations used to generate equivalent atoms:

#1 -x+1,-y+1,-z #2 x,y,z+1 #3 -x+1,-y,-z

#4 -x+1,-y+1,-z-1 #5 -x+1,-y,-z+1

The X-ray structure of **7** is shown in Figure 6 and selected bond lengths and angles for the complex are presented in Table 6. The structure consists of interlocked undulating polymeric ribbons running parallel to the *c* axis. Each manganese ion is coordinated to one bipy group, one bidentate carboxylate group and two further oxygen donors, one from each of two 1,3-carboxylate bridges (Figure 6). The diacids are each bonded to three different manganese ions, using one carboxylate as a bidentate ligand and the other as a bridging group. The manganese/isoph backbone of the polymer is shown in Figure 7.

Figure 8 shows the resulting honeycomb network, with $\pi$-stacking parallel to *a* and the polymer running parallel to *c*. The resulting lattice has channels parallel to *a* where the uncoordinated bipy molecules lie; these do not appear to have any significant interactions with the rest of the structure.

All of the novel complexes (**1-7**), the metal free ligands and simple salts of manganese were each tested for their ability to inhibit the growth of *Candida Albicans* (Table 7). Phthalic acid, isophthalic acid and the simple manganese salts are all essentially inactive against the pathogen. Furthermore, coordination of the acids to the metal (complexes **1** and **4**) does not result in any significant improvement in their activity. The uncoordinated bipyridine and again does not prevent the growth of the *Candida Albicans* and indeed the isophthalate complex incorporating the bipyridine ligand (complex **7**) only exhibits slight fungitoxic activity. The complexes incorporating the 1,10-phenanthroline ligand (complexes **2**, **3**, **5** and **6**) dramatically inhibit the growth of the fungus.

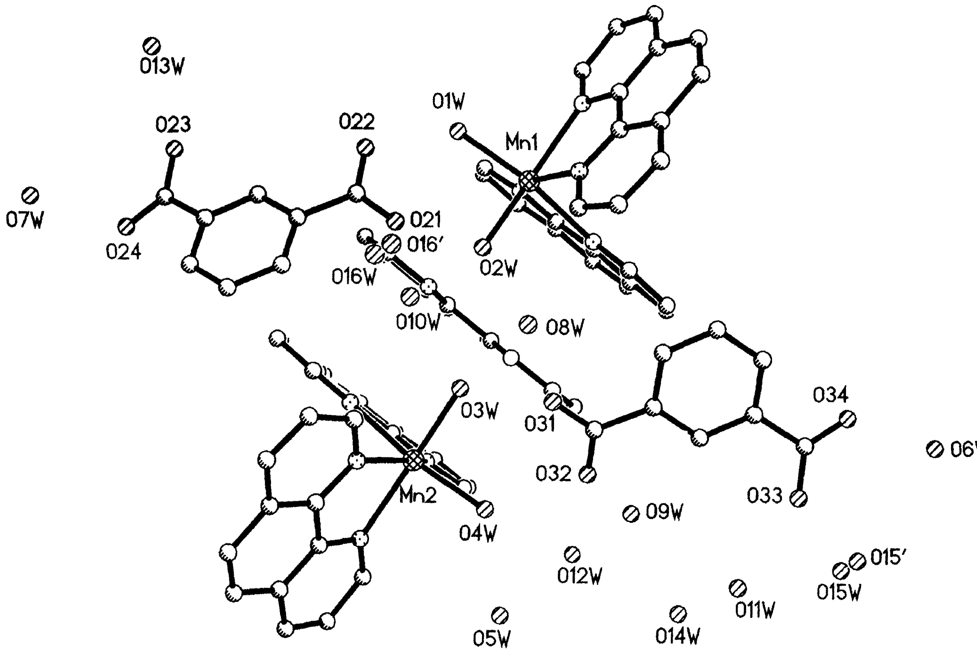

**Figure 3:** The asymmetric unit for $[Mn(phen)_2(H_2O)_2]_2(isoph)_2(phen)\cdot 12H_2O$ (**6**)

Table 4. Selected bond lengths [Å] and angles [°] for complex **6**.

| | | | |
|---|---|---|---|
| Mn(1)-O(2W) | 2.167(2) | Mn(2)-O(3W) | 2.149(2) |
| Mn(1)-N(2B) | 2.269(2) | Mn(2)-N(1C) | 2.258(2) |
| Mn(1)-N(1A) | 2.273(2) | Mn(2)-N(2D) | 2.272(2) |
| Mn(1)-N(1B) | 2.292(2) | Mn(2)-N(1D) | 2.288(2) |
| Mn(1)-N(2A) | 2.294(2) | Mn(2)-N(2C) | 2.313(2) |
| Mn(2)-O(4W) | 2.1289(19) | | |
| O(1W)-Mn(1)-O(2W) | 88.95(8) | O(3W)-Mn(2)-N(1D) | 164.84(8) |
| O(1W)-Mn(1)-N(2B) | 89.50(8) | N(1C)-Mn(2)-N(1D) | 91.46(8) |
| O(2W)-Mn(1)-N(2B) | 107.26(8) | N(2D)-Mn(2)-N(1D) | 73.23(9) |
| O(1W)-Mn(1)-N(1A) | 111.22(8) | O(4W)-Mn(2)-N(2C) | 163.78(8) |
| O(2W)-Mn(1)-N(1A) | 88.45(8) | O(3W)-Mn(2)-N(2C) | 89.13(8) |
| N(2B)-Mn(1)-N(1A) | 154.51(9) | N(1C)-Mn(2)-N(2C) | 72.66(8) |
| O(1W)-Mn(1)-N(1B) | 161.45(8) | N(2D)-Mn(2)-N(2C) | 86.50(8) |
| O(2W)-Mn(1)-N(1B) | 90.34(8) | N(1D)-Mn(2)-N(2C) | 96.45(8) |
| N(2B)-Mn(1)-N(1B) | 73.04(8) | | |
| N(1A)-Mn(1)-N(1B) | 87.28(8) | | |
| O(1W)-Mn(1)-N(2A) | 90.36(8) | | |
| O(2W)-Mn(1)-N(2A) | 159.53(8) | | |
| N(2B)-Mn(1)-N(2A) | 93.19(9) | | |
| N(1A)-Mn(1)-N(2A) | 72.77(9) | | |
| N(1B)-Mn(1)-N(2A) | 96.66(8) | | |
| O(4W)-Mn(2)-O(3W) | 87.81(7) | | |
| O(4W)-Mn(2)-N(1C) | 92.61(8) | | |
| O(3W)-Mn(2)-N(1C) | 103.66(8) | | |
| O(4W)-Mn(2)-N(2D) | 109.57(8) | | |
| O(3W)-Mn(2)-N(2D) | 93.15(8) | | |
| N(1C)-Mn(2)-N(2D) | 152.74(9) | | |
| O(4W)-Mn(2)-N(1D) | 90.53(8) | | |

Table 5. Hydrogen bonds for complex **6** [Å and °].

| D-H...A | d(D-H) | d(H...A) | d(D...A) | <(DHA) |
|---|---|---|---|---|
| O(1W)-H(1WA)...O(15W)#1 | 0.93 | 1.74 | 2.649(3) | 164.4 |
| O(1W)-H(1WB)...O(22) | 0.84 | 1.81 | 2.647(3) | 172.2 |
| O(2W)-H(2WA)...N(2E) | 0.94 | 1.94 | 2.837(3) | 160.4 |
| O(2W)-H(2WB)...O(21) | 0.88 | 1.87 | 2.732(3) | 165.0 |
| O(3W)-H(3WA)...N(1E) | 0.92 | 1.98 | 2.851(3) | 158.7 |
| O(3W)-H(3WB)...O(31) | 0.93 | 1.79 | 2.711(3) | 172.1 |
| O(4W)-H(4WA)...O(32) | 0.89 | 1.79 | 2.681(3) | 174.7 |
| O(4W)-H(4WB)...O(5W)#2 | 0.84 | 1.84 | 2.678(3) | 174.3 |
| O(5W)-H(5WA)...O(23) | 0.80 | 2.02 | 2.806(3) | 169.3 |
| O(5W)-H(5WB)...O(6W) | 0.96 | 1.71 | 2.671(3) | 172.0 |
| O(6W)-H(6WA)...O(34)#3 | 1.08 | 1.64 | 2.694(3) | 166.4 |
| O(6W)-H(6WB)...O(12W)#4 | 0.88 | 1.91 | 2.743(3) | 158.7 |
| O(7W)-H(7WA)...O(24) | 0.83 | 1.93 | 2.733(3) | 164.2 |
| O(7W)-H(7WB)...O(5W) | 0.93 | 2.00 | 2.825(3) | 147.4 |
| O(8W)-H(8WA)...O(31) | 0.92 | 1.87 | 2.753(3) | 160.8 |
| O(8W)-H(8WB)...O(10W) | 0.85 | 1.90 | 2.730(3) | 163.7 |
| O(9W)-H(9WA)...O(32) | 0.89 | 1.96 | 2.853(3) | 177.2 |
| O(9W)-H(9WB)...O(14W)#2 | 0.93 | 1.86 | 2.782(3) | 170.6 |
| O(10W)-H(0WA)...O(21) | 1.06 | 1.84 | 2.794(3) | 148.2 |
| O(10W)-H(0WB)...O(16W)#5 | 0.88 | 1.80 | 2.679(13) | 172.2 |
| O(10W)-H(0WB)...O(16')#5 | 0.88 | 2.11 | 2.906(5) | 150.2 |
| O(11W)-H(1WC)...O(33) | 0.97 | 1.79 | 2.726(3) | 162.4 |
| O(11W)-H(1WD)...O(22)#2 | 1.00 | 1.81 | 2.789(3) | 166.5 |
| O(12W)-H(2WC)...O(8W)#6 | 0.86 | 1.85 | 2.694(3) | 167.0 |
| O(12W)-H(2WD)...O(9W) | 0.82 | 1.98 | 2.771(3) | 163.3 |
| O(13W)-H(3WC)...O(32)#4 | 1.10 | 1.94 | 2.988(3) | 158.8 |
| O(13W)-H(3WD)...O(23) | 1.00 | 2.00 | 2.890(3) | 146.8 |
| O(14W)-H(4WC)...O(11W)#4 | 0.97 | 1.77 | 2.732(4) | 168.6 |
| O(14W)-H(4WD)...O(23) | 0.94 | 1.87 | 2.801(3) | 167.1 |
| O(15W)-H(5WC)...O(33)#3 | 0.88 | 1.89 | 2.647(3) | 142.0 |
| O(15W)-H(5WD)...O(7W) | 1.06 | 1.75 | 2.798(4) | 167.1 |
| O(16W)...............O(7W) | | | 2.92(3) | |
| O(16W)............ O(10W)#5 | | | 2.68(1) | |
| O(16')............ O(14W)#7 | | | 2.776(8) | |

Symmetry transformations used to generate equivalent atoms:

#1 x,-y+1/2,z-1/2 #2 x+1,y,z #3 x-1,-y+1/2,z+1/2 #4 x-1,y,z #5 -x,-y+1,-z+1

#6 -x+1,y-1/2,-z+1/2 #7 x, 1/2-y, 1/2+z

"Metal free" 1,10-phenanthroline showed very high anti-*Candida* activity and indeed this was only surpassed by the activity of the phthalate/1,10-phenanthroline complex **2**. In this case is thought that the powerful chelating ligand 1,10-phenanthroline is probably coordinating to metal ions (other than manganese) that are present in trace quantities in the growth medium and that it is these resulting metal-phenanthroline complexes that are responsible the observed anti-*candida* activity of the so called "metal free" phenanthroline. Studies are currently being carried out in our laboratories to examine this hypothesis.

In conclusion, it would appear that phthalic acid and isophthalic acid whether coordinated or uncoordinated to manganese do not posses any anti-*candida* properties. Furthermore the presence of the N, N-donor ligand bipyridine does not improve the activity of the isophthalate complex. The 1,10-phenanthroline molecule is a potent anti-*candida* agent and upon reaction with the phthalate and isophthalate complexes it yields compounds with comparable fungitoxic activity.

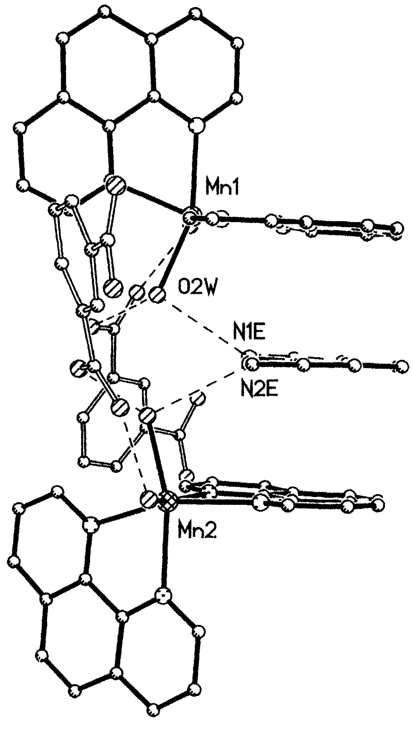

**Figure 4:** The π-π stacking of phenanthrolines in $[Mn(phen)_2(H_2O)_2]_2(isoph)_2(phen)\cdot 12H_2O$ (**6**)

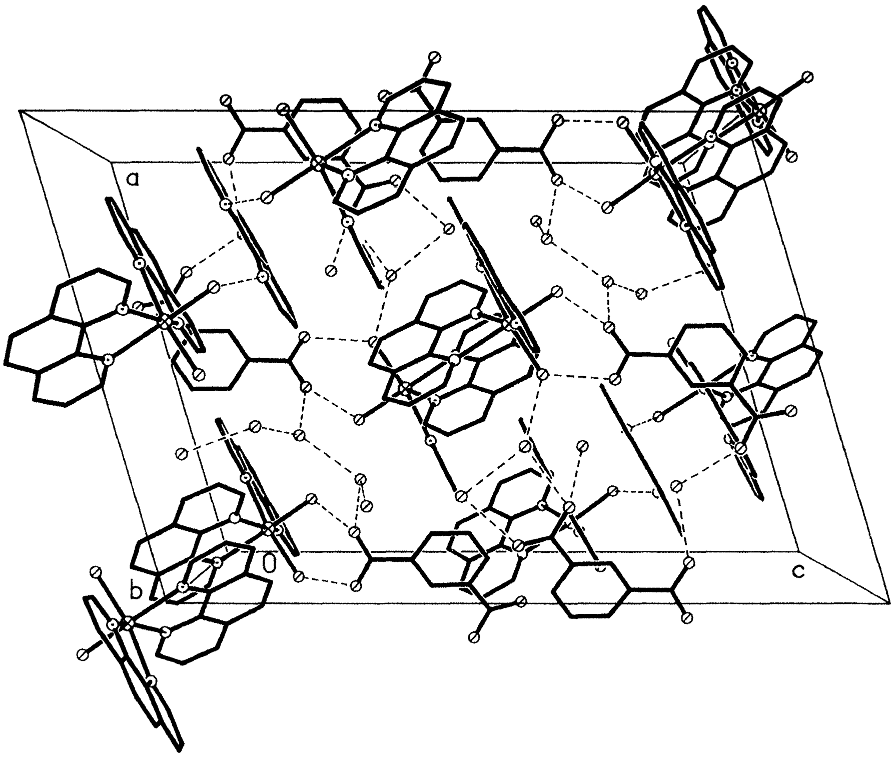

**Figure 5:** Packing diagram for $[Mn(phen)_2(H_2O)_2]_2(isoph)_2(phen)\cdot 12H_2O$ (**6**)

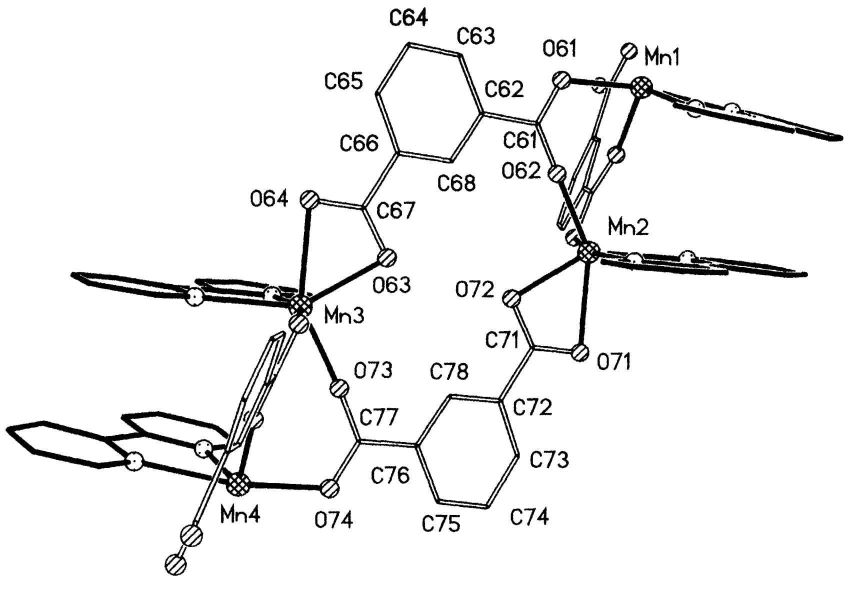

**Figure 6:** The structure of $[Mn(isoph)(bipy)]_4$ component of complex 7

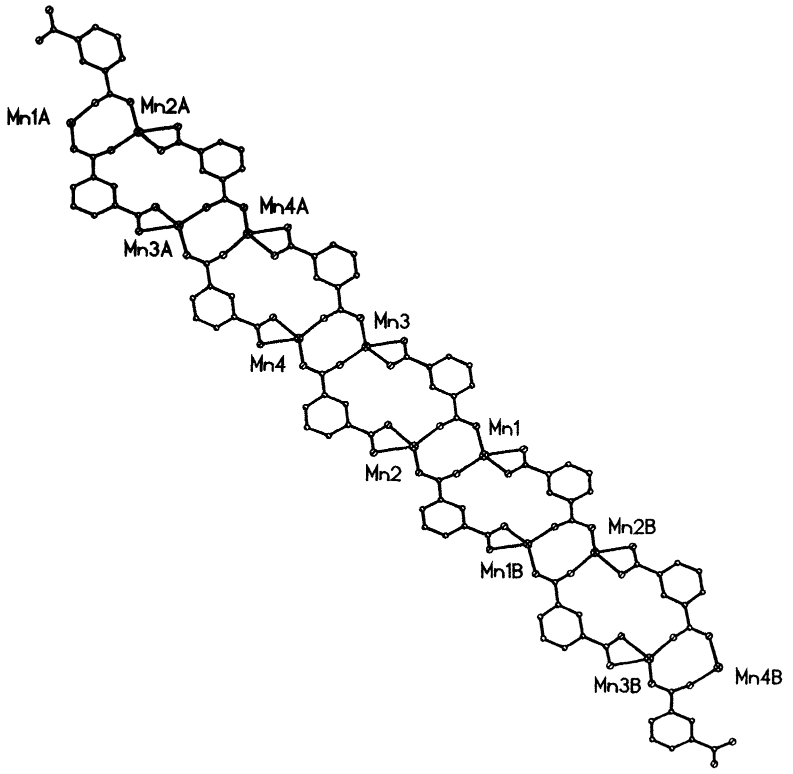

**Figure 7:** The manganese/isoph backbone of $\{[Mn(isoph)(bipy)]_4 \cdot 2.75bipy\}_n$ (7)

Table 6. Selected bond lengths [Å] and angles [°] for complex 7.

| | | | |
|---|---|---|---|
| Mn(1)-O(53) | 2.079(7) | Mn(3)-O(64) | 2.364(7) |
| Mn(1)-O(61) | 2.115(5) | Mn(3)-O(73) | 2.088(7) |
| Mn(1)-O(52)#1 | 2.193(6) | Mn(3)-O(81) | 2.100(5) |
| Mn(1)-N(11) | 2.274(6) | Mn(3)-N(31) | 2.289(7) |
| Mn(1)-N(12) | 2.277(7) | Mn(3)-N(32) | 2.291(6) |
| Mn(1)-O(51)#1 | 2.366(7) | Mn(4)-O(74) | 2.113(5) |
| Mn(2)-O(54) | 2.108(5) | Mn(4)-O(82) | 2.084(7) |
| Mn(2)-O(62) | 2.085(7) | Mn(4)-O(83)#2 | 2.188(6) |
| Mn(2)-O(72) | 2.181(6) | Mn(4)-N(42) | 2.273(6) |
| Mn(2)-N(21) | 2.290(6) | Mn(4)-N(41) | 2.282(6) |
| Mn(2)-N(22) | 2.294(6) | Mn(4)-O(84)#2 | 2.410(7) |
| Mn(2)-O(71) | 2.390(6) | | |
| Mn(3)-O(63) | 2.202(6) | | |
| O(53)-Mn(1)-O(61) | 96.2(2) | O(73)-Mn(3)-O(81) | 96.8(2) |
| O(53)-Mn(1)-O(52)#1 | 93.2(2) | O(73)-Mn(3)-O(63) | 91.9(2) |
| O(61)-Mn(1)-O(52)#1 | 106.5(2) | O(81)-Mn(3)-O(63) | 106.5(2) |
| O(53)-Mn(1)-N(11) | 116.1(3) | O(73)-Mn(3)-N(31) | 87.1(3) |
| O(61)-Mn(1)-N(11) | 93.0(2) | O(81)-Mn(3)-N(31) | 162.5(2) |
| O(52)#1-Mn(1)-N(11) | 143.0(3) | O(63)-Mn(3)-N(31) | 90.3(2) |
| O(53)-Mn(1)-N(12) | 84.9(3) | O(73)-Mn(3)-N(32) | 118.3(3) |
| O(61)-Mn(1)-N(12) | 162.7(3) | O(81)-Mn(3)-N(32) | 92.8(2) |
| O(52)#1-Mn(1)-N(12) | 90.7(3) | O(63)-Mn(3)-N(32) | 142.1(3) |
| N(11)-Mn(1)-N(12) | 71.2(3) | N(31)-Mn(3)-N(32) | 70.5(2) |
| O(53)-Mn(1)-O(51)#1 | 150.7(2) | O(73)-Mn(3)-O(64) | 149.4(2) |
| O(61)-Mn(1)-O(51)#1 | 95.8(2) | O(81)-Mn(3)-O(64) | 94.5(2) |
| O(52)#1-Mn(1)-O(51)#1 | 57.8(2) | O(63)-Mn(3)-O(64) | 57.6(2) |
| N(11)-Mn(1)-O(51)#1 | 89.8(2) | N(31)-Mn(3)-O(64) | 90.5(2) |
| N(12)-Mn(1)-O(51)#1 | 91.3(3) | N(32)-Mn(3)-O(64) | 89.4(2) |
| O(62)-Mn(2)-O(54) | 97.7(2) | O(82)-Mn(4)-O(74) | 98.6(2) |
| O(62)-Mn(2)-O(72) | 92.2(2) | O(82)-Mn(4)-O(83) | 91.4(2) |
| O(54)-Mn(2)-O(72) | 106.7(2) | O(74)-Mn(4)-O(83) | 107.6(2) |
| O(62)-Mn(2)-N(21) | 85.5(3) | O(82)-Mn(4)-N(42) | 86.2(3) |
| O(54)-Mn(2)-N(21) | 163.1(2) | O(74)-Mn(4)-N(42) | 161.6(2) |
| O(72)-Mn(2)-N(21) | 89.7(2) | O(83)-Mn(4)-N(42) | 89.9(2) |
| O(62)-Mn(2)-N(22) | 116.6(3) | O(82)-Mn(4)-N(41) | 117.7(3) |
| O(54)-Mn(2)-N(22) | 92.8(2) | O(74)-Mn(4)-N(41) | 91.4(2) |
| O(72)-Mn(2)-N(22) | 142.9(3) | O(83)#2-Mn(4)-N(41) | 142.7(3) |
| N(21)-Mn(2)-N(22) | 71.1(2) | N(42)-Mn(4)-N(41) | 70.9(2) |
| O(62)-Mn(2)-O(71) | 149.34(19) | O(82)-Mn(4)-O(84)#2 | 148.6(2) |
| O(54)-Mn(2)-O(71) | 95.6(2) | O(74)-Mn(4)-O(84)#2 | 95.0(2) |
| O(72)-Mn(2)-O(71) | 57.4(2) | O(83)#2-Mn(4)-O(84)#2 | 57.4(2) |
| N(21)-Mn(2)-O(71) | 89.7(2) | N(42)-Mn(4)-O(84)#2 | 89.7(2) |
| N(22)-Mn(2)-O(71) | 90.1(2) | N(41)-Mn(4)-O(84)#2 | 90.0(2) |

Symmetry transformations used to generate equivalent atoms: #1, 1-x, 1-y, -z; #2, 3-x, 1-y, 1-z

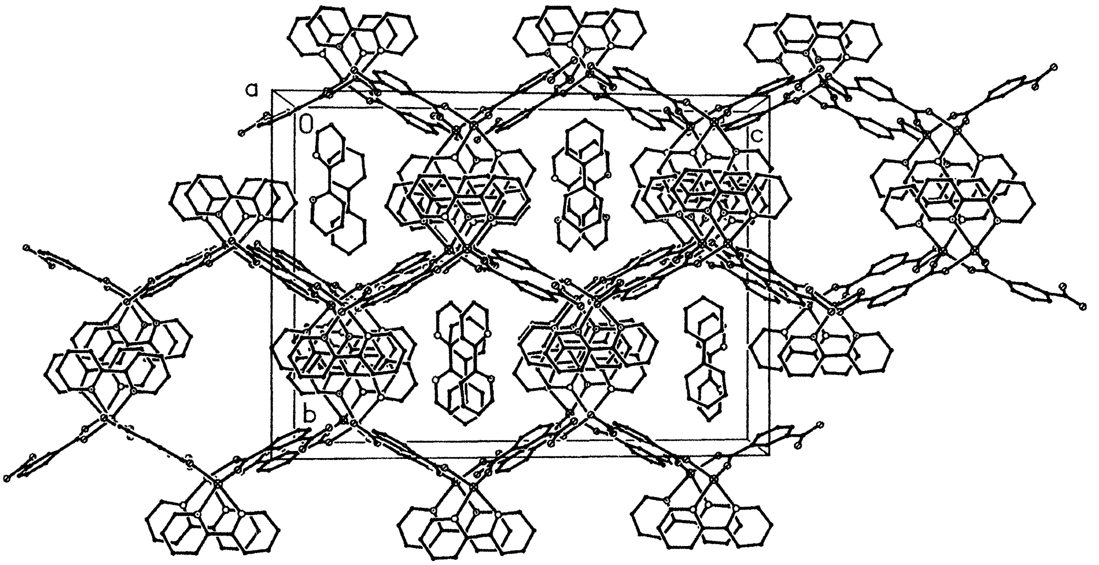

**Figure 8:** The packing diagram for the structure of {[Mn(isoph)(bipy)]$_4$·2.75bipy}$_n$ (7)

**Table 7:** Anti-*candida* activity. *

| Test Compound | %Cell Growth |
|---|---|
| | **Isolate** |
| Control | 100 |
| Phthalic acid | 100 ± 8 |
| Isophthalic acid | 108 ± 12 |
| 1,10-phenanthroline | 23 ± 2 |
| 2,2'-bipyridine | 114 ± 6 |
| $MnCl_2$ | 110 ± 5 |
| $Mn(NO_3)_2$ | 133 ± 7 |
| $Mn(OAc)_2$ | 117 ± 10 |
| $Mn(SO_4)$ | 101 ± 9 |
| [Mn(ph)]·0.5$H_2O$ **1** | 113 ± 7 |
| [Mn(ph)(phen)]·2($H_2O$) **2** | 19 ± 2 |
| [Mn(ph)(phen)$_2$($H_2O$)]·4$H_2O$ **3** | 30 ± 2 |
| [Mn(isoph)]·2$H_2O$ **4** | 131 ± 15 |
| [Mn$_2$(isoph)$_2$(phen)$_3$]·4$H_2O$ **5** | 26 ± 3 |
| [Mn(phen)$_2$($H_2O$)$_2$]$_2$(isoph)$_2$(phen)·12$H_2O$ **6** | 28 ± 2 |
| {[Mn(isoph)(bipy)]$_4$·2.75$H_2O$}$_n$ **7** | 90 ± 10 |

- The compounds were tested at concentrations of 10 μg $cm^{-3}$ of aqueous RPMI medium. The complexes **5** and **7** were insoluble in water and were used as suspensions. Yeast cells were grown for 24h at 37° C. Results are presented as % cell growth and the effectiveness of the compounds are compared to the growth of the control (no compound added).

**Acknowledgements**

M. Devereux acknowledges the late Noel O'Reilly(DIT) and Iris Swift(DIT) for technical assistance, and V Leon acknowledges the SRD and Seed funding schemes (DIT/EU) for financial support. This work has been carried out (in part) within the structures of the Facility for Optical Characterisation and Spectroscopy (FOCAS), funded by the Irish Government Programme for Research in Third Level Institutions.

**References**

1. T. J. Walsh, J. W. Hiemenz and E.Anaissie, *Infect. Dis. Clin. North Am.*, 1996, **29**, 365.
2. A. H. Groll, *J. Infect.*, 1996, **33**, 23.
3. G. Y. Minamoto and A. S. rosenberg, *Med. Clin. North Am., 1997,* **81,** 381.
4. A. H. Groll, A. J. De lucca and T. J. Walsh, *Trends in Microbiology*, 1998, **6**, 117.
5. A. L Barry and S. D. Brown, *Antimicrob. Agents Chemother.*, 1996, **40**, 1948.

6. See for example: M. Petric, F. Pohleven, I. Turel, P. Segedin, A. White and D. Williams, *Polyhedron*, 1998, **17**, 255.
7. M. Geraghty, V. Sheridan, M. McCann, M. Devereux and V. McKee, *Polyhedron*, 1999, **18**, 2931.
8. M. Devereux, G.M. McCann, V. Leon, M. Geraghty, V. McKee and Jan Wikaira, *Polyhedron*, 2000, **19**, 1205.
9. M. Geraghty, M. McCann, M. Devereux, F. Cronin, M. Curran and V. McKee, *Metal Based Drugs*, 1999, **6**, 41.
10. M. Geraghty, M.McCann, M. Devereux and V. McKee, *Inorg. Chim. Acta.*, 1999, **293**, 160.
11. M. Geraghty, F. Cronin, M. Devereux and M. McCann, *Biometals*, 2000, **13**, 1.
12. G. M. Sheldrick, SHELXL-97, University of Göttingen, 1997.
13. NCCLS (National Committee for Clinical Laboratory Standards), reference method for broth dilution antifungal susceptibility testing of yeasts; proposed standard. NCCLS, Villanova, Pa. NCCLS publication (1992) M27-P

**Received: November 7, 2000 – Accepted: December 12, 2000**
**Received in revised camera-ready format: march 1, 2001**

